# Oral Hygiene Status, Salivary and Microbiological Parameters Among Visually Impaired and Normal-Sighted Children After Specialized Oral Health Education: An Interventional Study

**DOI:** 10.7759/cureus.56304

**Published:** 2024-03-17

**Authors:** Apurva P Deshpande, Anil V Ankola, Roopali M Sankeshwari, Mehul A Shah, Laxmi Kabra, Atrey J Pai Khot, Ram Surath Kumar

**Affiliations:** 1 Department of Public Health Dentistry, KLE Vishwanath Katti Institute of Dental Sciences, KLE Academy of Higher Education and Research, Belagavi, IND; 2 Department of Public Health Dentistry, Goa Dental College and Hospital, Goa, IND; 3 Department of Public Health Dentistry, Bharati Vidyapeeth (Deemed to be University) Dental College and Hospital, Pune, IND

**Keywords:** health education, oral health, oral hygiene, salivary parameters, special child, visually impaired

## Abstract

Aim and objectives

To assess oral hygiene status and salivary and microbiological parameters among 12 to 15-year-old visually impaired and normal-sighted children before and after oral health education (OHE).

Methodology

An interventional study was conducted among 25 visually impaired children (Group A) and 25 normal-sighted children (Group B) in the age range of 12 to 15 years. Simple random sampling was used to select the study participants. A questionnaire was designed to record socio-demographic data and the dietary habits of the children on pre-decided days. The oral hygiene practices and the Decayed Missing Filled Teeth (DMFT) index were recorded, and salivary physicochemical parameters for all the selected children were evaluated, followed by saliva collection for microbial analysis. After baseline assessment, the Audio-Tactile Performance technique for Group A and the animated visual performance technique for Group B children were used to impart OHE. Periodic assessments of salivary parameters were conducted at one-month and three-month intervals. Unpaired T test/Mann-Whitney U test, repeated measures analysis of variance (ANOVA)/Friedman test, followed by Bonferroni’s post hoc test were carried out to determine the difference between and within groups, respectively. All statistical tests were performed at a significance level of 5%.

Results

Group A demonstrated a greater change in salivary pH (6.20 ± 0.41 to 6.96 ± 0.20), salivary buffering capacity (5.80 ± 0.82 to 7.20 ± 0.65), and *Streptococcus mutans* count (9.36 ± 0.41 to 8.7 ± 0.45 x 10^4^ CFU/mL) when compared to Group B. Group B demonstrated a greater *Lactobacillus acidophilus* count reduction (7.96 ± 0.66 to 7.50 ± 0.64 x 10^4^ CFU/mL) when compared to Group A.

Conclusion

The appropriate use of specialized OHE holds particular significance in the improvement of oral hygiene status and salivary parameters, along with a reduction in the bacterial count in both visually impaired children and normal-sighted children.

## Introduction

The World Health Organization (WHO) has conceptualized that oral health plays a vital role in improving the performance of individuals. One of the crucial factors for having good oral health is knowledge about oral hygiene practices [1]. Knowledge of oral health, if given during the formative years of the child, remains with them for a lifetime [2]. However, a major challenge lies in educating and treating children with vision impairments due to communication skill problems and alternative pedagogical requirements.

Vision impairment has an unfavorable effect on the routine life of an individual. WHO estimates that 36 million people around the globe are visually impaired, and 11.7 million of them are in India [3]. According to the latest survey, there are 206,523 visually impaired children in India. It accounts for 72.9% of all the visually challenged children in Southeast Asia who are below 15 years of age [4].

Early signs of gingivitis, dental caries, or cavities can typically be identified through vision and go unnoticed in visually impaired children [5]. One of the intrinsic factors of clinical importance is saliva, which serves as a mirror of the body’s health. Various physicochemical and microbiological properties of saliva have an impact on oral hygiene status. These parameters have been established as sensitive indicators in caries prediction models and play a significant role in untangling information about the risk of developing oral diseases [6]. Impairment in vision acts as a barrier to an individual's ability to engage with everyday tasks, so exploring specialized oral health education techniques is of special significance. Dental health is one of the greatest unattended health needs of visually impaired children. Hence, appropriate oral health education by healthcare professionals would educate these children about oral hygiene practices, dental diseases, the role of a proper diet, and their adeptness regarding primary preventive strategies, making them self-sufficient.

The impact of specially curated oral health education (OHE) on the salivary and microbiological parameters and oral hygiene status of children with special health care needs and normal-sighted children has not been evaluated extensively. Literature quotes various OHE intervention techniques for imparting knowledge to enlighten visually impaired children. One such technique is the Audio-Tactile Performance (ATP) method [7-9]. Therefore, the present study was planned to assess oral hygiene status and salivary and microbiological parameters among 12 to 15-year-old visually impaired and normal-sighted children before and after OHE in Belagavi, Karnataka.

## Materials and methods

Study design and ethical considerations

An interventional study was conducted among 12 to 15-year-old visually impaired children and normal-sighted children from July 2021 to November 2021. A list of all the government schools for normal children and a list of special schools in Belagavi, Karnataka, India, were obtained from the Deputy Director of Public Instructions (DDPI). One school in each category was then selected randomly by the lottery method. Ethical approval was obtained from the Institutional Ethics and Research Committee (Ethical Code No. 1307).

Piloting of the study and sample size estimation

Before conducting the study, the training and calibration of the principal investigator were conducted to record the Decayed Missing Filled Teeth (DMFT) index and salivary physicochemical parameters. Intra-examiner variability was calculated using Kappa statistics and was found to be 0.94. A pilot study was conducted among 10 normal-sighted children and 10 visually impaired children to assess the flaws and feasibility of the study. The following formula was used for sample size estimation: n = (Z1-α/2 + Z1-β)^2 (SD1^2 + SD2^2) / (x1 - x2)^2, where Z1-α/2 corresponds to the two-tailed significance level (1.96 for .05), Z1-β corresponds to power (.84 = 80% power), SD corresponds to the standard deviation, and X1 - X2 corresponds to effect size. A sample size of 50 was calculated as per the study conducted by Shetty et al. (2014), taking a 95% confidence interval, 80% power of the study, and a 10% dropout rate [10]. The study followed Consolidated Standards of Reporting Trials (CONSORT) guidelines. However, randomization was not possible as the study involved two different study groups.

Inclusion and exclusion criteria

All the participants, aged between 12 and 15 years, were screened for inclusion and exclusion criteria at both schools. Visually impaired children having visual acuity ranging from 6/60 to 1/60 and normally sighted children with completely erupted index teeth, children who gave assent, and children’s parents/guardians who gave written consent were included in the study. Children with any systemic diseases or with signs and symptoms of COVID-19, who had taken antibiotics within one month before or during the study and used any chemical mode of plaque control during the study in the past month, who were medically compromised, with intellectual disability, and with multiple conditions like visual impairment along with other conditions were excluded.

Participant recruitment and collection of sample

A total of 57 visually impaired children and 97 normal-sighted children aged 12-15 years underwent the screening procedure, of whom 34 and 58 fulfilled the inclusion and exclusion criteria, respectively. Of these, 25 participants in each group were selected randomly using a computer-generated table of random numbers, making a total sample size of 50. Group A is comprised of visually impaired children, and Group B is comprised of normal-sighted children.

A questionnaire was prepared to record socio-demographic data, dietary habits, oral hygiene practices, the Decayed Missing Filled Teeth (DMFT) index, and physicochemical and microbiological parameters of saliva. Kuppuswamy Socio-economic classification was used to assess the socio-economic status.

The present study was conducted during the COVID-19 pandemic. A standard operating protocol was followed as the study involved saliva collection and analysis. The schools were visited on the scheduled dates, and the DMFT index and salivary physicochemical parameters (pH and buffering capacity) for all the selected children were evaluated, followed by saliva collection for microbial analysis. After the salivary parameters’ evaluation, the plaque index, gingival index, and DMFT index were recorded [11,12]. A Type III examination was performed under natural lighting conditions.

The collection of saliva was conducted between 8 a.m. and 11 a.m. to maintain the circadian cycle. Children were asked not to eat or drink any beverages except water and not to perform any particular oral hygiene except rinsing the mouth with drinking water. The children were given drinking water and asked to rinse their mouths. Five minutes after the mouth rinse, saliva collection was carried out, which was followed by salivary parameter evaluation. Salivary samples for laboratory analysis were transported in 1 ml of “reduced transport fluid” (RTF) transport media in sterile Eppendorf tubes. The children were also asked not to cough up mucus during saliva collection [13,14]. A whole unstimulated salivary sample was collected in a disposable syringe. The principal investigator was trained, and all the laboratory procedures were performed under the microbiologist's supervision.

Physicochemical parameters of saliva

Salivary pH was analyzed using color-indicating pH strips from Merck Company with a specific range. One drop of saliva was placed on the strip. The color change was noted and compared with the pH color scale provided with the strip.

Buffering capacity: 0.5 mL of saliva was added to 1.5 mL of 0.005 molarity hydrochloric acid (HCL). A pH strip was dipped into this solution. The color change was noted and compared with the pH color scale provided with the strip. The pH noted was the buffering capacity of saliva.

Microbiological parameters of saliva

Saliva was collected in a disposable syringe, and 1 mL of saliva was injected into 1 mL of “reduced transport fluid” (RTF) transport media. The saliva samples of all the subjects were identified by code number during the period of sample collection and processing. The same code was used for a particular subject during the subsequent sample collection. The sample was transported to the laboratory immediately after collection and cultured on the same day. The processing was conducted at the Basic Science Research Laboratory, KLE Academy of Higher Education & Research (KAHER), and the Department of Microbiology, Jawaharlal Nehru Medical College (JNMC), KAHER, and the mean was considered the final value.

Samples were vortexed into a uniform mix of saliva using a vortex mixer. The first dilution of the sample was 1:2 (1 mL of saliva sample into 1 mL of RTF). The next dilution consisted of a dilution of saliva (100 µl of the above mixture) into 4.5 ml of thioglycolate broth. Hence, the dilution became 1:50. Taking into account the previous dilution (1:2) and the next dilution (1:50), a dilution of 1:100 was considered, of which 10 µl was plated on individual media, Mitis Salivaris agar for *Streptococcus mutans* and Rogosa SL for *Lactobacillus acidophilus*, using an inoculation loop. The plates were incubated for 48 hours at 37^o^C in a 5-10% carbon dioxide (CO_2_) jar. After 48 hours, colony characteristics were studied, and the number of “colony-forming units” of *Streptococcus mutans* (CFU/mL) and *Lactobacillus acidophilus* (CFU/mL) in saliva was determined. They were identified at the genus level by gram staining.

Oral health evaluation

The gingival index was used to describe the clinical severity of gingival inflammation, and the plaque index was used to assess the amount of dental plaque. All the children were clinically assessed according to the 2013 WHO Oral Health Assessment form [15]. Following the baseline examination, OHE was given to both groups.

Administration of appropriate specialized OHE

For Group A, the ATP technique was used to impart OHE to visually impaired children. In this method, the children were informed verbally about the importance of teeth and the method of brushing (Audio), and then they were made to feel the teeth on a large-sized model (Tactile) followed by brushing on the model using the “Modified Bass Method” with assistance (Performance). This was repeated until the children could perform with ease. In addition, all the children were explained about the formation of oral diseases and diet counseling and were enlightened regarding the golden rules to maintain good oral hygiene and health.

For Group B, the animated visual performance technique was used to impart OHE to normal-sighted children. In this method, the children were shown a customized animated video with cartoon characters describing the development of oral diseases, oral hygiene practices, the relationship between oral health and general health, golden rules, and appropriate brushing techniques. After the video, all the children were asked to demonstrate the brushing technique on the tooth model until they mastered the technique.

The clinical parameters (plaque and gingival index), salivary physicochemical (pH and buffering capacity), and microbial (*Streptococcus mutans* and *Lactobacillus acidophilus*) parameters were evaluated at one month and three months from baseline examination.

Statistical analysis

Data was entered in Microsoft Excel 2013, Version 15 (Microsoft Corporation, Redmond, Washington, United States), and analyzed using IBM SPSS Statistics for Windows, Version 21 (released 2012; IBM Corp., Armonk, New York, United States). Descriptive statistics were used to calculate frequencies, percentages, and mean values. The association between the factors and groups when both are qualitative was determined using the chi-square test. Unpaired T test/Mann-Whitney U test, repeated measures analysis of variance (ANOVA), Friedman test, followed by Bonferroni’s post hoc test were carried out to determine the difference between and within groups, respectively. All statistical tests were performed at a significance level of 5% (p ≤ 0.05).

## Results

The mean age of the participants was 13.16 ± 0.96 and 12.6 ± 0.63 in Group A (visually impaired children) and Group B (normal children), respectively. The demographic characteristics of the study population are presented in Table 1.

**Table 1:** Demographic characteristics of the study population The statistical test employed: ¥One-way ANOVA and #Chi-square test; the significance threshold was set at p ≤.05, indicating a statistically significant result. ANOVA: analysis of variance

Demographic characteristics		Group A	Group B	Chi-square value	p-value
Age in years^¥^	Mean ± SD	13.1 ± 0.96	12.6 ± 0.63	9.919	0.007*
Gender^#^	Male	14 (56%)	13 (52%)	0.0810	0.7770
	Female	11 (44%)	12 (48%)		
Socioeconomic status^#^	Upper	0 (0%)	1 (4%)	1.3670	0.7130
	Upper Middle	2 (8%)	3 (12%)		
	Lower Middle	13 (52%)	11 (44%)		
	Upper Lower	10 (40%)	10 (40%)		
	Lower	0 (0%)	0 (0%)		

A statistically significant difference was observed between the groups regarding their dietary habits, as indicated by statistical significance in the type of diet (p = 0.017), sweet consumption time (p < .05), and the consistency of sweets (p = 0.005). However, no significant difference was observed in the type of staple diet consumed (Table 2).

**Table 2:** Distribution of visually impaired children (Group A) and normal children (Group B) according to food habits All values are expressed as frequency with percentages (in parentheses); The statistical test used: Chi-square test; level of significance: *p ≤ 0.05 is considered statistically significant.

Food habits					Statistical analysis	
Question	Response	Group A N (%)	Group B N (%)	Total N (%)	Chi-square	p-value
What is your staple diet?	Wheat	15 (60%)	16 (64%)	31 (62%)	0.085	0.771
	Rice	10 (40%)	9 (36%)	19 (38%)		
Type of diet?	Vegetarian	13 (52%)	4 (16%)	17 (34%)	5.704	0.017*
	Mixed	12 (48%)	21 (84%)	33 (66%)		
How many times did you eat sweets in the last 24 hours?	None	1 (4%)	12 (48%)	13 (26%)	18.732	0.001*
	Once	5 (20%)	6 (24%)	11 (22%)		
	Twice	5 (20%)	5 (20%)	10 (20%)		
	Thrice	11 (44%)	1 (4%)	12 (24%)		
	> 3times	3 (12%)	1 (4%)	4 (8%)		
When were the sweets eaten?	During meals	5 (20%)	5 (20%)	10 (20%)	13.83	0.003*
	In between meals	8 (32%)	3 (12%)	11 (22%)		
	Both	11 (44%)	5 (20%)	16 (32%)		
	Not applicable	1 (4%)	12 (48%)	13 (26%)		
What is the consistency of the sweets?	Solid	12 (48%)	6 (24%)	18 (36%)	12.78	0.005*
	Liquid	8 (32%)	4 (16%)	12 (24%)		
	Sticky	4 (16%)	3 (12%)	7 (14%)		
	Not applicable	1 (4%)	12 (48%)	13 (26%)		
	Total	25 (100%)	25 (100%)	50 (100%)		
Total		25 (100%)	25 (100%)	50 (100%)		

The mean DMFT score for permanent dentition in Group A was 5.44 ± 3.00, whereas in Group B it was 3.20 ± 2.15, and it was found to be statistically significant. Conversely, the mean dmft score for deciduous dentition in Group A was 0.84 ± 1.51, and in Group B it was 0.48 ± 0.69, which was found to be statistically insignificant (Table 3). 

**Table 3:** Intergroup comparison of visually impaired children (Group A) and normal children (Group B) with respect to caries experience DMFT/dmft – Decayed Missing Filled teeth; All values are expressed as Mean ± Standard deviation (SD); The statistical test used: Unpaired T-test; level of significance: *p ≤ 0.05 is considered statistically significant.

Variable	Group A Mean ± SD	Group B Mean ± SD	p-value
DMFT	5.44 ± 3.00	3.20 ± 2.15	0.004*
dmft	0.84 ± 1.51	0.48 ± 0.69	0.296

There was no statistically significant distinction found between the two groups in terms of gingival and plaque scores at baseline, one month, and three months (Table 4).

**Table 4:** Intergroup comparison of visually impaired children (Group A) and normal children (Group B) with clinical parameters at different time intervals All values are expressed as Mean ± Standard deviation (SD); Different Greek symbols (α, β, γ) indicate a significant difference within the group at various time intervals (in the column); The statistical test used: Bonferroni post hoc method following a significant ‡Repeated measures ANOVA and †Unpaired t-test; level of significance: *p ≤ 0.05 is considered statistically significant. ANOVA: analysis of variance

Clinical parameter	Time point	Group		Statistical analysis
		Group A Mean ± SD	Group B Mean ± SD	p-value^†^
Gingival Index	Baseline	1.35 ± 0.57^α^	1.32 ± 0.77^α^	0.9304
	1 month	1.00 ± 0.44^β^	1.02 ± 0.61^β^	0.9536
	3 months	0.58 ± 0.39^γ^	0.54 ± 0.47^γ^	0.4434
p-value^‡^		0.0001*	0.0001*	
Plaque index	Baseline	1.56 ± 0.73^α^	1.38 ± 0.73^α^	0.3567
	1 month	1.08 ± 0.58^β^	0.95 ± 0.61^β^	0.3368
	3 months	0.59 ± 0.42^γ^	0.46 ± 0.40^γ^	0.3177
p-value^‡^		0.0001*	0.0001*	

**Figure 1:**
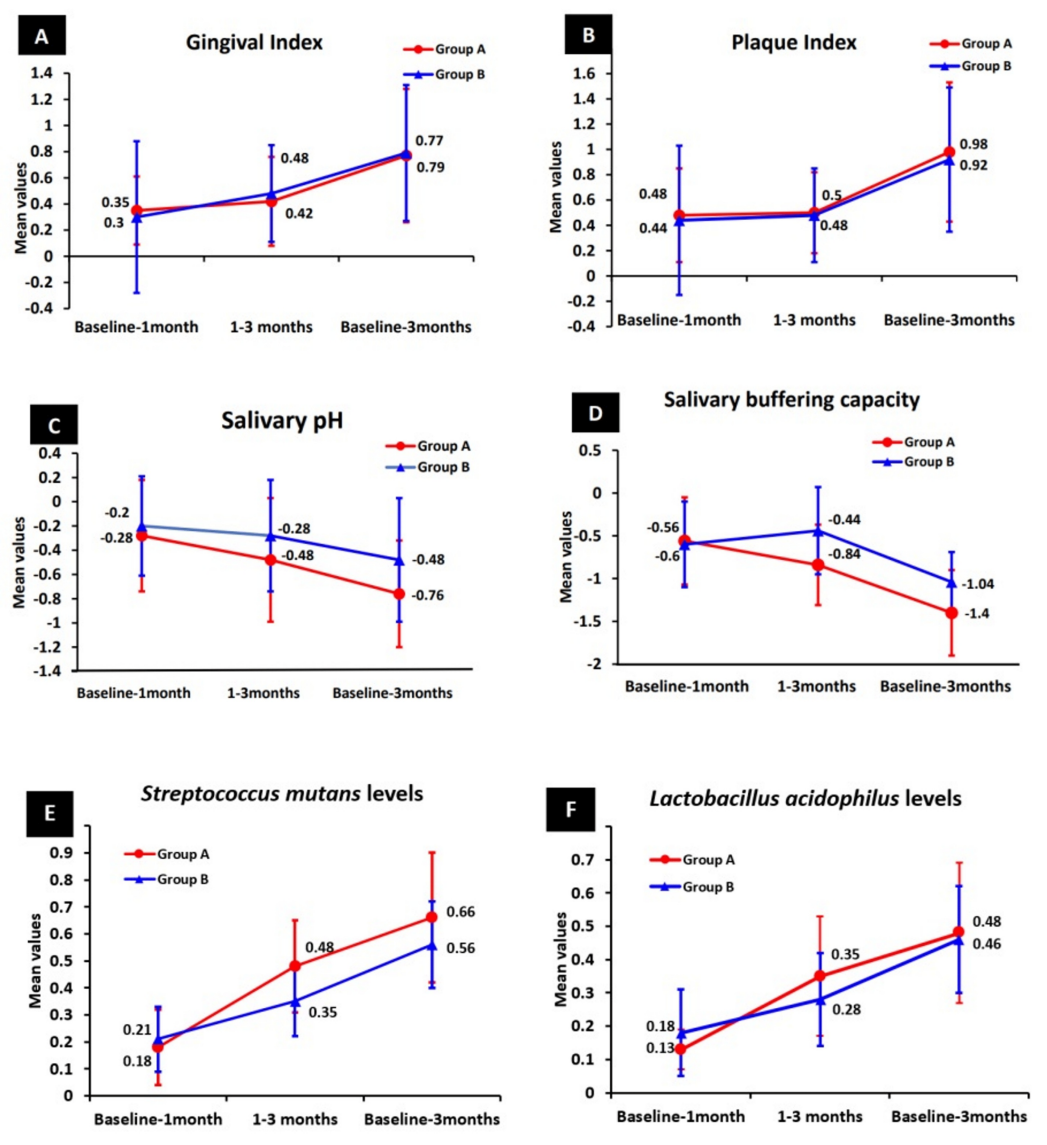
Mean change in (A) gingival score, (B) plaque score, (C) salivary pH, (D) buffering capacity, and (E, F) microbiological parameters among visually impaired and normal-sighted children

The change in mean reduction of gingival score in group A was 0.35 from baseline to one month, 0.42 from one month to three months, and 0.77 from baseline to three months. However, the mean reduction change in group B was 0.30 from baseline to one month, 0.48 from one month to three months, and 0.79 from baseline to three months. A similar trend was observed for the plaque score. Group B demonstrated a greater gingival index (GI) and plaque index (PI) score reduction when compared to Group A. A statistically significant difference was observed at all time intervals in both groups (p < 0.05). Mean changes in GI and PI scores, salivary pH and buffering capacity, and microbiological parameters among visually impaired and normal-sighted children are depicted in Figure 1.

The mean salivary pH score at baseline was 6.20 ± 0.41 in Group A, while it was 6.56 ± 0.58 in Group B and was statistically significant (p = 0.015). However, there was no significant difference in salivary pH at one-month (p = 0.061) and three-month follow-ups (p = 0.163) between the groups. The mean salivary buffering capacity score was found to be statistically significant at baseline (p = 0.006) and one month (p = 0.005). The difference between the mean salivary scores (salivary pH and buffering capacity) of both groups at the various time intervals was found to be statistically significant.

The mean counts of *Streptococcus mutans* and *Lactobacillus acidophilus* exhibited a significant decrease at the one-month and three-month follow-ups (p = 0.0001) in both groups. Group A demonstrated a greater change in salivary pH, salivary buffering capacity, and counts of *Streptococcus mutans* and *Lactobacillus acidophilus* compared to Group B (Table 5).

**Table 5:** Intergroup comparison of visually impaired children (Group A) and normal children (Group B) with salivary parameters at different time intervals All values are expressed as Mean ± Standard deviation (SD); Different Greek symbols (α, β, γ) indicate a significant difference within the group at various time intervals (in the column); The statistical test used: Bonferroni post hoc method following a significant ‡Repeated measures ANOVA / #Friedman test and †Unpaired t-test / ¥Mann-Whitney U-test; level of significance: *p ≤ 0.05 is considered statistically significant.

Parameter	Time point	Group		Statistical analysis
		Group A Mean ± SD	Group B Mean ± SD	p-value^†^
Salivary parameter				
Salivary pH	Baseline	6.20 ± 0.41^α^	6.56 ± 0.58^α^	0.0148*
	1 month	6.48 ± 0.51^β^	6.76 ± 0.52^β^	0.0612
	3 months	6.96 ± 0.20^γ^	7.04 ± 0.20^γ^	0.1638
p-value^‡^		0.0001*	0.0001*	
Salivary buffering capacity	Baseline	5.80 ± 0.82^α^	6.40 ± 0.65^α^	0.0059*
	1 month	6.36 ± 0.76^β^	7.00 ± 0.76^β^	0.0046*
	3 months	7.20 ± 0.65^γ^	7.44 ± 0.58^γ^	0.1741
p-value^‡^		0.0001*	0.0001*	
Microbiological parameter				p-value^¥^
Salivary Streptococcus mutans levels (*10^4^ CFU/ml)	Baseline	9.36 ± 0.41^α^	8.58 ± 0.68^α^	0.0001*
	1 month	9.18 ± 0.44^ β^	8.37 ± 0.67^ β^	0.0001*
	3 months	8.70 ± 0.45^ γ^	8.02 ± 0.65^ γ^	0.0001*
p-value^#^		0.0001*	0.0001*	
Salivary Lactobacillus acidophilus levels (*10^4^ CFU/ml)	Baseline	8.79 ± 0.35^α^	7.96 ± 0.66^α^	0.0001*
	1 month	8.66 ± 0.35^β^	7.78 ± 0.62^β^	0.0001*
	3 months	8.31 ± 0.38^γ^	7.50 ± 0.64^γ^	0.0001*
p-value^#^		0.0001*	0.0001*	

## Discussion

The application of appropriate health education in oral health is a well-acknowledged initiative in averting oral diseases. It brings out new behaviors that will promote and improve individual health [16]. A child’s schooling period is considered to be the most appropriate time to impart OHE for the prevention of oral health problems. It has been documented that oral health status and knowledge regarding oral health may significantly improve if the health promotion of children is carried out comprehensively and interestingly. The use of the ATP technique and animations has gained popularity while giving education in oral health to visually impaired children and normal school children, respectively. Various studies and systematic reviews have shown remarkable development in oral hygiene status after delivering OHE using these techniques [9,17-20]. Hence, these methods were implemented to impart education to both groups.

Children in the age group of 12 to 15 years were selected to carry out this study as they are most susceptible to caries as well as gingival and periodontal diseases owing to hormonal influence, changes in dietary habits, and lifestyle [21]. The majority of visually impaired children consumed a vegetarian diet, while the majority of normal-sighted children consumed a mixed diet. A positive relationship was obtained in a study conducted by Lashkari et al., which highlighted the fact that vegetarians have an increased risk of dental caries compared with non-vegetarians [22]. Similar results were seen in our study: visually impaired children had higher mean DMFT scores when compared to normal-sighted children.

Sweet consumption was observed more in visually impaired children when compared to normal-sighted children in the last 24 hours. Moreover, a greater percentage of visually impaired children consumed sweets during meals as well as in between meals. It has been postulated from the findings of the Vipeholm dental caries study that the increase in the frequency of consumption of sugar between meals was associated with an increase in the occurrence of dental caries [23]. Similarly, the present study delineates a higher dental caries prevalence in visually impaired children than in normally sighted children who have a greater history of sugar consumption.

In the current study, the mean DMFT scores were 5.4 ± 3.0 and 3.2 ± 2.2 in visually impaired and normal-sighted children, respectively. The mean dmft score was 0.8 ± 1.5 in visually impaired children, while it was 0.5 ± 0.7 in normal-sighted children. These findings were in agreement with the previous research by Prashanth et al. [24]. and Solanki et al. [25], where a higher caries prevalence was noted in visually impaired children. It may be attributed to the lack of awareness and knowledge regarding oral hygiene practices in visually impaired children and the high intake of sugar observed in the current study.

A significant difference was observed at different time intervals in both groups concerning salivary physicochemical and microbiological parameters. Salivary pH and buffering capacity in normal-sighted children were higher than in visually impaired children at baseline. Moreover, microbiological parameters like *Streptococcus mutans* and *Lactobacillus acidophilus* were more common in visually impaired children when compared to normal-sighted children at baseline. These findings highlight the fact that visually impaired children are more susceptible to dental caries and poor oral hygiene when compared to normal-sighted children. However, post-educational improvements in salivary pH and buffering capacity and a reduction in *Streptococcus mutans* counts were seen in visually impaired children when compared to normal-sighted children. Furthermore, a maximum decrease in *Lactobacillus acidophilus *count was observed in normal-sighted children when compared to visually impaired children.

Oral health messages through interesting educational media can aid in the retention of new knowledge or the acquisition of a new skill. Very few studies have been done to date where animated videos are used to deliver oral health education, and future studies should incorporate such methods to assess their effectiveness.

These findings also signify that appropriate OHE helped children in both groups improve their salivary physicochemical and microbiological parameters. Furthermore, it can be noted that visually impaired children were more cautious about their oral health and needed appropriate guidance to practice correct oral hygiene practices. Similar results were seen in various observational studies [13,26]. The current literature is devoid of interventional studies assessing the effect of OHE on salivary parameters and oral hygiene status among visually impaired and normal-sighted children.

Overall, the salivary parameters of both visually impaired and normal-sighted children were unsatisfactory at baseline, and more inferior among visually impaired children. This may be attributed to the fact that during lockdown due to the COVID-19 pandemic, children were at home, which would have led to frequent snacking and a lack of OHE to follow correct oral hygiene practices.

The current study revealed that, by rendering appropriate integration of OHE interventions and active participation of children, there was an improvement in salivary parameters in both groups. To the best of our knowledge, this extensive study is the first of its kind where salivary physicochemical and microbiological parameters are compared before and after specialized OHE.

Strengths

The present study had no dropouts. As per our knowledge, this extensive study is the first of its kind where salivary physicochemical and microbiological parameters, gingival scores, and plaque scores are compared before and after specialized oral health education techniques in visually impaired and normal children.

The present study was conducted during the ongoing COVID-19 pandemic, where appropriate oral health education would help them take appropriate oral hygiene precautions and would aid in reducing salivary oral pathogens. In addition to oral hygiene instructions, both visually impaired and normal children were educated about standard precautions to be taken during the COVID-19 pandemic to prevent its transmission and spread.

Children with special health care needs often lack choices that would assist them in improving their oral hygiene. The current study gave them the chance that visually impaired children deserve.

Limitation

The study was conducted with a limited follow-up period.

Prospects and recommendations

OHE and supervised oral hygiene practices should be incorporated into the regular curriculum for visually impaired children. More qualitative and exploratory studies are necessary to assess the long-term sustainability of educational interventions. Appropriate reinforcement plays a major role in imparting OHE to special children. Future studies should be planned with a longer follow-up period.

## Conclusions

The overall improvement in salivary pH, buffering capacity, and reduction in *Streptococcus mutans* count was greater in visually impaired children, while the reduction in *Lactobacillus acidophilus* count was greater in normal-sighted children post-intervention. Thus, the appropriate use of specialized OHE holds particular significance in the improvement of oral hygiene status as well as salivary and microbiological parameters in both visually impaired children and normal-sighted children.
