# Correction: Oral Hygiene Status, Salivary and Microbiological Parameters Among Visually Impaired and Normal-Sighted Children After Specialized Oral Health Education: An Interventional Study

**DOI:** 10.7759/cureus.c198

**Published:** 2024-11-08

**Authors:** Apurva P Deshpande, Anil V Ankola, Roopali M Sankeshwari, Mehul A Shah, Laxmi Kabra, Atrey J Pai Khot, Ram Surath Kumar

**Affiliations:** 1 Department of Public Health Dentistry, KLE Vishwanath Katti Institute of Dental Sciences, KLE Academy of Higher Education and Research, Belagavi, IND; 2 Department of Public Health Dentistry, Goa Dental College and Hospital, Goa, IND; 3 Department of Public Health Dentistry, Bharati Vidyapeeth (Deemed to be University) Dental College and Hospital, Pune, IND

This article has been corrected due to an incorrect author list. The name of the fourth author was incorrectly listed as Mahantesh B. Nagmoti, but the correct fourth author is Mehul A. Shah. The author list has now been updated to reflect the correct authorship.

